# Community engagement in sexual health and uptake of HIV testing and syphilis testing among MSM in China: a cross-sectional online survey

**DOI:** 10.7448/IAS.20.01/21372

**Published:** 2017-04-03

**Authors:** Tiange P. Zhang, Chuncheng Liu, Larry Han, Weiming Tang, Jessica Mao, Terrence Wong, Ye Zhang, Songyuan Tang, Bin Yang, Chongyi Wei, Joseph D. Tucker

**Affiliations:** ^a^ SESH Research Team at the School of Medicine, University of North Carolina at Chapel Hill, Guangzhou, China; ^b^ Stritch School of Medicine, Loyola University Chicago, Maywood, IL, USA; ^c^ University of North Carolina at Chapel Hill Project-China, Guangzhou, China; ^d^ School of Medicine, University of North Carolina at Chapel Hill, Chapel Hill, NC, USA; ^e^ Guangdong Provincial Center for Skin Diseases and Sexually Transmitted Infections Control, Guangzhou, China; ^f^ Department of Epidemiology and Biostatistics, University of California San Francisco, San Francisco, CA, USA

**Keywords:** HIV, men who have sex with men (MSM), community engagement, HIV testing, syphilis testing, China, low- and middle-income countries (LMICs)

## Abstract

**Introduction**: HIV and syphilis testing rates remain low among men who have sex with men (MSM) in low- and middle-income countries (LMICs). Community engagement has been increasingly used to promote HIV testing among key populations in high-income countries, often in settings with stronger civil society. This study aimed to assess socio-demographic, behavioural, and community engagement factors associated with HIV and syphilis testing among MSM in China.

**Methods**: MSM ≥16 years old who had condomless sex in the past three months were recruited nationwide to complete a cross-sectional online survey in November 2015. Data were collected on socio-demographics, sexual behaviours, HIV testing, syphilis testing, and community engagement in sexual health. We defined community engagement in sexual health using six items assessing awareness and advocacy of sexual health programmes. The underlying factor structure of a 6-item community engagement scale was determined through exploratory factor analysis. Univariate and multivariable logistic regressions identified correlates of HIV and syphilis testing.

**Results**: 1189 MSM were recruited. 54% (647/1189) of men had ever tested for HIV and 30% (354/1189) had ever tested for syphilis. Factor analysis suggested three levels of community engagement (minimal, moderate, and substantial) and this model explained 79.5% of observed variance. A quarter (26%, 312/1189) reported none to minimal engagement, over one half (54%, 644/1189) reported moderate engagement, and a fifth (20%, 233/1189) reported substantial engagement. Multivariable logistic regression showed that MSM with greater community engagement in sexual health were more likely to have ever tested for HIV (substantial vs. no engagement: aOR 7.91, 95% CI 4.98–12.57) and for syphilis (substantial vs. no engagement: aOR 5.35, 95% CI 3.16–9.04).

**Conclusions**: HIV and syphilis testing are suboptimal among MSM in China. Community engagement may be useful for promoting testing in China and should be considered in intervention development and delivery. Further research is needed to better understand the role of LMIC community engagement in HIV interventions.

## Introduction

Men who have sex with men (MSM) worldwide confront barriers that limit access to key HIV prevention services [1–3]. HIV testing plays an essential role in the HIV care continuum but has failed to adequately reach many MSM in low- and middle-income countries (LMICs) [4]. Global weighted estimates indicate that only 31% of MSM in LMICs were tested for HIV [5]. Knowledge of HIV status is vital to linking patients to timely treatment and preventing further transmission [6,7]. Early diagnosis permits initiation of antiretroviral treatment, which limits both disease progression and transmission [6,8]. Additionally, knowledge of seropositive HIV status is linked to lower HIV risk behaviour and lower risk of transmission [7,8].

In China, the government has significantly expanded surveillance efforts among MSM in response to the rapid spread of HIV in this key population [9]. Yet, several systematic reviews suggest that still only half of MSM have ever tested for HIV [10–12]. In addition, syphilis test uptake is poor in China [11], which is concerning given the rise in HIV and syphilis co-infections among Chinese MSM [13,14]. The Chinese Centers for Disease Control and Prevention (CDC) offer free HIV testing at voluntary counselling and testing sites [15]. However, Chinese MSM face major psychosocial and structural barriers to testing, including fear of stigma and discrimination from providers, concerns over confidentiality, and inconvenience of testing sites [16–19]. Greater understanding of testing behaviours among MSM is needed to inform public health strategies.

MSM have significantly contributed to the HIV response through community leadership and participation [20]. Community engagement is defined by the US CDC as “the process of working collaboratively with groups of people affiliated by proximity, interests, or situations with respect to issues affecting their wellbeing” [21]. Among the range of community engagement activities, interventions that engage MSM in the promotion and advocacy of sexual health are increasingly used to expand HIV testing [20,22,23]. For example, a peer-led HIV intervention that engaged community members through social media led to a three-fold increase in HIV testing among MSM [22]. Community engagement in sexual health may create positive social norms towards testing among MSM, thereby reducing fear of stigma and increasing willingness to test [24–26].

Most studies on community engagement have been conducted in settings with a strong civil society, where non-governmental and not-for-profit organizations operate independently from both the state and the market [27]. Less is known about the important relationship between community engagement and HIV testing in LMICs with constrained civil society, like China. Civil society organizations (CSOs), the wide array of non-governmental and not-for-profit organizations that have a presence in public life [27], have had a growing yet still limited role in China’s HIV response due to the restrictive legal environment, inadequate funding, and limited personnel [28]. Further investigation of community engagement using locally appropriate measures may be useful for improving community-level HIV testing interventions in China.

Increasing HIV testing uptake and frequency is particularly important among MSM living in LMICs. Yet, HIV testing research on this key population has been limited in LMICs [29,30]. Insights into community engagement and other factors associated with HIV testing and syphilis testing can facilitate the development of MSM-led, community-level interventions. The purpose of this study was to describe community engagement in sexual health and other potential correlates of HIV testing and syphilis testing among MSM in China.

## Methods

### Study participants and setting

We recruited MSM to participate in a nationwide online survey in November 2015. The survey link was promoted on major social networking platforms in China. These platforms included the following: Danlan.org, a large lesbian, gay, bisexual, and transgender (LGBT) web portal (a specifically designed website that serves as a single point of access for information); Weibo, a microblogging website, a web service that allows the user to post and share short messages with other users of the service; BlueD, a mobile dating app; and WeChat, a mobile messaging app. Clicking on the link directed participants to the survey hosted on Qualtrics (Provo, Utah). Participants were considered eligible if they had anal sex with men at least once during their lifetime, had condomless anal and/or vaginal sex in the past three months, were assigned male gender at birth, and were at least 16 years of age (age of legal consent in China). The survey questions stemmed from an extensive literature review and were piloted among 150 MSM [31].

### Measures

We defined HIV testing as self-reported history of ever receiving a HIV test. We defined syphilis testing as self-reported history of ever receiving a syphilis test. Information was collected about HIV testing and syphilis testing. We also asked about HIV testing frequency, which was categorized as less than once every two years, once a year, once every six months, once every three months, or monthly.

Participants were also asked about socio-demographic information, behavioural information, and community engagement in sexual health. Several variables were assessed for association with testing history, including age, education, income, sexual orientation, number of male sex partners in the past three months, and community engagement in sexual health. Sexual orientation was defined as gay, bisexual, or heterosexual and others for analysis.

We defined “community engagement in sexual health” as awareness and advocacy of sexual health among community members (abbreviated in analyses as “engagement”). This measure aimed to capture a subset of community engagement activities that relate to sexual health and take place among community members. To construct a single scale measure, six survey items (Additional file 1) were first adapted from existing community engagement literature [25,32–34] and piloted among 150 Chinese MSM before the online survey. These metrics included ever discussed HIV/sexually transmitted infection (STI) testing or sexual health online, awareness of ongoing MSM sexual health events, ever helped organize MSM sexual health campaigns, ever volunteered to help provide MSM sexual health services, ever encouraged someone to test for HIV/STIs, and ever accompanied someone to a HIV/STI testing facility.

### Statistical analysis

First, we conducted descriptive analysis of socio-demographic and behaviour characteristics, HIV testing and syphilis testing history, and community engagement in sexual health metrics.

The Kaiser-Meyer-Olkin measure of sampling adequacy was considered (overall 0.70) in assessing correlation matrices’ suitability for factor analysis. Bartlett’s test of sphericity verified that more than one factor was required (chi-square: 158.7, *p* < 0.001). We conducted an exploratory factor analysis [35] using principal components extraction and promax rotation to identify the underlying factor structure of the six community engagement in sexual health items. The optimal number of factors was determined using a scree test, Kaiser’s criterion, and Horn’s parallel analysis [35]. Loading variables were determined by extracting all items with loadings >0.70. No items loaded on more than one factor. Cronbach’s alpha coefficient and the Kuder–Richardson 20 for dichotomous data were calculated to measure the internal consistency of the items. We then examined the association between testing and these factors using multivariable logistic regression.

Three separate sets of multivariable logistic regression were conducted, each based on a different outcome variable. First, all survey participants were classified based on history of ever having tested for HIV. Those ever having tested for HIV were compared to those never tested for HIV. Second, those ever having tested for HIV were classified into subgroups based on frequency of HIV testing. Frequent HIV testing was defined as those having tested at least once every six months, a cutoff adopted from the widely used recommendation by US CDC, which recommends MSM to test for HIV once every 3–6 months [36]. Then, frequent HIV testers were compared to infrequent HIV testers. Third, all survey participants were classified based on history of ever having tested for syphilis. Those ever having tested for syphilis were compared to those never tested for syphilis. Univariate logistic regression was conducted to identify variables associated with the outcome variable in each analysis set. Socio-demographic variables pre-selected to adjust for potential confounders included age, education, and income. Multivariable logistic regression was then conducted, adjusting for age, education, and income in the model. All analysis was conducted using SAS version 9.4 (Cary, NC).

### Ethical review

Survey participants agreed to informed consent. This study was approved by the Institutional Review Boards of the Guangdong Provincial Center for Skin Diseases and STI Control, the University of North Carolina at Chapel Hill, and the University of California San Francisco.

## Results

### Study participants

Overall, the survey link was clicked on 7892 times. Of the 1579 individuals who met inclusion criteria and agreed to the online informed consent, 1189 (75%) completed the survey. Socio-demographic and behavioural characteristics of participants are detailed in Table 1. Participant distribution across the four major geographic regions in China is similar to the population distribution across the four regions (Additional file 4).

**Table 1.** Socio-demographic and behavioural characteristics of MSM in China, 2015 (*n* = 1189)

Characteristic	Total *N* (%)	No engagement *N* (%)	Minimal engagement *N* (%)	Moderate engagement *N* (%)	Substantial engagement *N* (%)
**Community engagement in sexual health ^a^**	1189 (100)	165 (14)	147 (12)	644 (54)	233 (20)
**Age (years)**					
≤30	989 (83)	140 (85)	126 (86)	535 (83)	188 (81)
>30	200 (17)	25 (15)	21 (14)	109 (17)	45 (19)
**Marital status with female**					
Never married	991 (83)	133 (81)	123 (84)	546 (85)	189 (81)
Ever married	198 (17)	32 (19)	24 (16)	98 (15)	44 (19)
**Student status**					
Yes	431 (36)	51 (31)	61 (42)	229 (36)	90 (39)
No	758 (64)	114 (69)	86 (58)	415 (64)	143 (61)
**Education ^b^**					
Vocational college or Lower	698 (59)	97 (59)	97 (66)	369 (57)	135 (58)
Four-year college or graduate degree	491 (41)	68 (41)	50 (34)	275 (43)	98 (42)
**Annual income (USD)**					
<2700	330 (28)	44 (27)	57 (39)	164 (25)	65 (28)
2700–5500	312 (26)	47 (28)	37 (25)	174 (27)	54 (23)
5501–9200	333 (28)	53 (32)	33 (22)	178 (28)	69 (30)
9201–15000	139 (12)	14 (9)	17 (12)	76 (12)	32 (14)
>15000	75 (6)	7 (4)	3 (2)	52 (8)	13 (5)
**Sexual orientation**					
Gay	835 (70)	105 (64)	100 (68)	464 (72)	166 (71)
Bisexual	308 (26)	51 (31)	39 (27)	157 (24)	61 (26)
Heterosexual and others	46 (4)	9 (5)	8 (5)	23 (4)	6 (3)
**Number of male sex partners in the past 3 months**					
0–1	597 (50)	76 (46)	80 (54)	332 (52)	109 (47)
Multiple ^c^	592 (50)	89 (54)	67 (46)	312 (48)	124 (53)
**Have primary male sex partner in the past 3 months^d^**					
Yes	840 (71)	103 (62)	94 (64)	467 (73)	176 (76)
No	349 (29)	62 (38)	53 (36)	177 (27)	57 (24)
**Have casual male sex partner in the past 3 months ^e^**					
Yes	606 (51)	82 (50)	79 (54)	328 (51)	117 (50)
No	583 (49)	83 (50)	68 (46)	316 (49)	116 (50)
**Ever had sex with female**					
Yes	335 (28)	46 (28)	43 (29)	176 (27)	70 (30)
No	854 (72)	119 (72)	104 (71)	468 (73)	163 (70)
**Ever tested for HIV**					
Yes, Frequent HIV tester ^f^	278 (23)	9 (5)	10 (7)	169 (26)	90 (39)
Yes, Infrequent HIV tester	369 (31)	31 (19)	24 (16)	238 (37)	75 (32)
No	542 (46)	125 (76)	113 (77)	236 (37)	68 (29)
**Ever tested for syphilis**					
Yes	354 (30)	22 (13)	18 (12)	209 (33)	105 (45)
No	835 (70)	143 (87)	129 (88)	435 (67)	128 (55)
**Perceived importance of community participation in developing sexual health campaigns**					
Very important	560 (47)	43 (26)	52 (35)	306 (48)	159 (68)
Important	370 (31)	65 (39)	56 (38)	202 (31)	47 (20)
Neither important or not important	171 (14)	30 (118)	22 (15)	98 (15)	21 (9)
Slightly important	56 (5)	15 (9)	10 (7)	27 (4)	4 (2)
Not important	32 (3)	12 (7)	7 (5)	11 (2)	2 (1)

a: “Community engagement in sexual health” is defined as awareness and advocacy of sexual health among community members; b: “Education” is defined as the highest degree one achieved; c: number of “multiple male sex partner” range from 2 to 92; d: “primary male sex partner” is defined as a person that the participant have sex regularly with and/or have an emotional commitment to; e: “casual male sex partner” is defined as a person that the participant have sex with and do not have an emotional commitment to; f: “frequent HIV tester” is defined as having tested at least once every 6 months.

### Characteristics and testing history

The majority (83%, 989/1189) of participants were under 30 years of age (Mean = 25, Range: 16–58), were never married (83%, 991/1189), identified as gay (70%, 835/1189), and were not students (64%, 758/1189). Over half did not have a 4-year college degree (59%, 698/1189), and had an annual income less than 5500 USD (54%, 642/1189). Most participants (71%, 840/1189) had a primary male sex partner in the past 3 months and had never had sex with women (72%, 854/1189). Roughly half of participants (51%, 606/1189) had casual male sex partners in the past three months. Roughly half (54%, 647/1189) had ever tested for HIV and a quarter (23%, 278/1189) tested for HIV frequently. Roughly a third (30%, 354/1189) had ever tested for syphilis.

### Exploratory factor analysis

The factor analysis identified three latent factors within the 6-item engagement scale (Additional file 2). These factors accounted for 79.5% of the observed variance. The three factors were named minimal, moderate, and substantial engagement. Cronbach’s alpha for the combination of the six items was 0.67 and the Kuder–Richardson coefficient was 0.65.

Each MSM participant was categorized as having no, minimal, moderate, or substantial engagement based on responses to the six items. No engagement indicated lack of involvement in all six items. Minimal engagement items included: question 1 “Ever discussed HIV/STI testing or sexual health online,” and question 2 “Aware of ongoing sexual health community events among MSM.” Moderate engagement items included: question 5 “Ever encouraged someone else to test for HIV/STIs,” and question 6 “Ever accompanied someone to a testing facility to test for HIV/STIs.” Substantial engagement items included: question 3 “Ever helped organize a sexual health campaign among MSM,” and question 4 “Ever volunteered to help provide sexual health services among MSM.” Participants who met criteria for more than one level were grouped in the highest level of engagement. For example, a participant who had encouraged someone to test for HIV/STIs (question 5, moderate) and helped organize a sexual health campaign among MSM (question 3, substantial) would be grouped as substantial engagement.

### Community engagement in sexual health

Among the participants, 14% reported no engagement (165/1189), 12% reported minimal engagement (147/1189), over one-half reported moderate engagement (54%, 644/1189), and a fifth reported substantial engagement (20%, 233/1189) (Table 1). Roughly half of participants perceived community participation in developing sexual health campaigns to be “very important” (47%, 560/1189) (Table 1). Responses to individual questions in the 6-item engagement scale are shown in Additional file 3.

Multivariable models adjusted for potential confounders (age, education, income) are presented for having ever tested for HIV (Table 2), for having frequently tested for HIV (Table 3), and for having ever tested for syphilis (Table 4). In general, greater level of community engagement in sexual health was associated with higher likelihood of testing. For having ever tested for HIV, moderate engagement (*p* < 0.001) and substantial engagement (*p* < 0.001) were significant correlates in the multivariable model. Substantial engagement (aOR 7.91, 95% CI 4.98–12.57) had a greater increase in likelihood of HIV testing than moderate engagement (aOR 5.52, 95% CI 3.71–8.22) when both were compared to no engagement. Similarly, for having frequently tested for HIV, moderate engagement (*p* < 0.017) and substantial engagement (*p* < 0.001) were significant correlates, with substantial engagement (aOR 4.30, 95% CI 1.92–9.63) having a greater increase in likelihood of frequent HIV testing than moderate engagement (aOR 2.56, 95% CI 1.18–5.53). Finally, for having ever tested for syphilis, moderate engagement (*p* < 0.001) and substantial engagement (*p* < 0.001) were again significant correlates, with substantial engagement (aOR 5.35, 95% CI 3.16–9.04) having a greater increase in likelihood of syphilis testing than moderate engagement (aOR 3.07, 95% CI 1.89–4.98). Adjusting additionally for “having multiple male sex partners in the past 3 months” in the multivariable model did not change the results (Additional file 5). Univariate and multivariable models regressing testing history on each of the 6 items in the engagement scale are summarized in Additional file 3.

**Table 2.** Univariate and multivariable models of HIV testing among MSM in China, 2015 (*n* = 1189)

Characteristic	Ever tested for HIV (%)	Never tested for HIV (%)	*P*	uOR (95% CI)	*P*	aOR (95% CI)
**Age (years)**						
≤30	519 (52.5)	470 (47.5)		Reference		
>30	128 (64)	72 (36)	0.003	1.61 (1.18, 2.21)	0.209	1.26 (0.88, 1.82)
**Education**						
Vocational college or Lower	339 (48.6)	359 (51.4)		Reference		
Four-year college or graduate degree	308 (62.7)	183 (37.3)	<0.001	1.78 (1.41, 2.26)	<0.001	1.76 (1.25, 2.28)
**Annual income (USD)**						
<2700	149 (45.2)	181 (54.8)		Reference		
2700–5500	165 (52.9)	147 (47.1)	0.050	1.36 (1.00, 1.86)	0.053	1.40 (0.99, 1.98)
5501–9200	187 (56.2)	146 (43.8)	0.005	1.56 (1.15, 2.11)	0.013	1.54 (1.09, 2.16)
9201–15000	92 (66.2)	47 (33.8)	<0.001	2.38 (1.57, 3.59)	0.002	2.05 (1.30, 3.24)
>15000	54 (72)	21 (28)	<0.001	3.12 (1.81, 5.41)	0.018	2.09 (1.13, 3.85)
**Sexual orientation**						
Gay	480 (57.5)	355 (42.5)	0.118	1.61 (0.89, 2.92)	0.127	1.61 (0.87, 2.97)
Bisexual	146 (47.4)	308 (52.6)	0.824	1.07 (0.58, 2.00)	0.949	0.98 (0.52, 1.85)
Heterosexual and others	21 (45.7)	46 (54.3)		Reference		
**Number of male sex partners in the past 3 months**						
0–1	301 (50.4)	296 (49.6)		Reference		
Multiple	346 (58.4)	246 (41.6)	0.006	1.38 (1.10, 1.74)	0.015	1.34 (1.06, 1.69)
**Community engagement in sexual health**						
No engagement	40 (24.2)	125 (75.8)		Reference		
Minimal engagement	34 (23.1)	113 (76.9)	0.818	0.94 (0.56, 1.59)	0.932	1.02 (0.60, 1.74)
Moderate engagement	277 (57.8)	166 (42.2)	<0.001	5.40 (3.66, 7.98)	<0.001	5.52 (3.71, 8.22)
Substantial engagement	346 (71.5)	138 (28.5)	<0.001	7.58 (4.81, 11.95)	<0.001	7.91 (4.98, 12.57)

Multivariable analysis controlled for age, education, annual income.

**Table 3.** Univariate and multivariable models of frequent/infrequent HIV tester among MSM in China, 2015 (*n* = 646)

Characteristic	Frequent HIV testers (%)	Infrequent HIV testers (%)	*P*	uOR (95% CI)	*P*	aOR (95% CI)
**Age (years)**						
≤30	224 (43.2)	295 (56.8)	0.896	Reference		
>30	54 (42.5)	73 (57.5)		0.97 (0.66, 1.44)	0.689	1.09 (0.71, 1.67)
**Education**						
Vocational College or Lower	142 (41.9)	197 (58.1)		Reference		
Four-year university or graduate school	136 (44.3)	171 (55.7)	0.536	1.10 (0.81, 1.51)	0.320	1.18 (0.85, 1.64)
**Annual income (USD)**						
<2700	69 (46.3)	80 (53.7)		Reference		
2700–5500	69 (41.8)	96 (58.2)	0.424	0.83 (0.53, 1.30)	0.416	0.83 (0.52, 1.31)
5501–9200	82 (44.6)	102 (55.4)	0.653	0.91 (0.59, 1.40)	0.614	0.89 (0.57, 1.40)
9201–15000	46 (50)	46 (50)	0.577	1.16 (0.69, 1.95)	0.931	1.10 (0.60, 1.76)
>15000	12 (22.6)	41 (77.4)	0.003	0.34 (0.17, 0.70)	0.002	0.29 (0.14, 0.63)
**Sexual orientation**						
Gay	211 (44.1)	268 (55.9)	0.168	1.97 (0.75, 5.16)	0.172	1.97 (0.75, 5.20)
Bisexual	61 (41.8)	85 (58.2)	0.253	1.79 (0.66, 4.89)	0.257	1.80 (0.65, 4.94)
Heterosexual and others	6 (28.6)	15 (71.4)		Reference		
**Number of male sex partners in past 3 months**						
0–1	118 (39.2)	183 (61.0)		Reference		
Multiple	160 (46.4)	185 (53.6)	0.66	1.34 (0.98, 1.84)	0.069	1.34 (0.98, 1.85)
**Community engagement in sexual health**						
No engagement	9 (22.5)	31 (77.5)		Reference		
Minimal engagement	10 (29.4)	24 (70.6)	0.499	1.44 (0.50, 4.09)	0.549	1.38 (0.48, 3.94)
Moderate engagement	83 (36.6)	144 (63.4)	0.022	2.45 (1.14, 5.27)	0.017	2.56 (1.18, 5.53)
Substantial engagement	176 (51.0)	169 (49.0)	0.001	4.13 (1.85, 9.23)	<0.001	4.30 (1.92, 9.63)

Multivariable analysis controlled for age, education, and annual income.

**Table 4.** Univariate and multivariable models of syphilis testing among MSM in China, 2015 (*n* = 1189)

Characteristic	Ever tested for syphilis (%)	Never tested for syphilis (%)	*P*	uOR (95% CI)	*P*	aOR (95% CI)
**Age (years)**						
≤30	267 (27.0)	722 (73.0)		Reference		
>30	87 (43.5)	113 (56.5)	<0.001	2.08 (1.52, 2.85)	0.009	1.59 (1.13, 2.26)
**Education level**						
Vocational college or Lower	188 (26.9)	510 (73.1)		Reference		
Four-year college or graduate degree	166 (33.8)	325 (66.2)	0.012	1.39 (1.08, 1.77)	0.077	1.28 (0.97, 1.67)
**Annual income (USD)**						
<2700	65 (19.7)	265 (80.3)		Reference		
2700–5500	93 (29.9)	218 (70.1)	0.38	1.29 (0.73, 2.27)	0.006	1.71 (1.17, 2.50)
5501–9200	100 (30.1)	232 (69.9)	<0.001	2.14 (1.29, 3.57)	0.012	1.62 (1.11, 2.36)
9201–15000	58 (41.7)	81 (58.3)	<0.001	2.16 (1.29, 3.62)	<0.001	2.42 (1.53, 3.82)
>15000	36 (48.0)	39 (52.0)	<0.001	3.76 (2.22, 6.38)	0.001	2.58 (1.44, 4.60)
**Sexual orientation**						
Gay	262 (31.4)	573 (68.6)	0.05	0.46 (0.21, 1.00)	0.039	2.30 (1.04, 5.08)
Bisexual	84 (27.3)	224 (72.7)	0.159	0.56 (0.25, 1.25)	0.199	1.71 (0.76, 3.87)
Heterosexual and others	8 (17.4)	38 (82.6)		Reference		
**Number of male sex partners in past 3 months**						
0–1	160 (26.8)	437 (73.2)		Reference		
Multiple	194 (32.8)	398 (67.2)	0.026	1.33 (1.04, 1.71)	0.09	1.25 (0.97, 1.61)
**Community engagement in sexual health**						
No engagement	22 (13.3)	143 (86.7)		Reference		
Minimal engagement	18 (12.2)	129 (87.8)	0.774	0.91 (0.47, 1.77)	0.928	0.97 (0.49, 1.90)
Moderate engagement	112 (28.5)	281 (71.5)	<0.001	3.12 (1.94, 5.04)	<0.001	3.07 (1.89, 4.98)
Substantial engagement	202 (41.7)	282 (58.3)	<0.001	5.33 (3.18, 8.95)	<0.001	5.35 (3.16, 9.04)


Multivariable analysis controlled for age, education, and annual income.

## Discussion

Despite extensive efforts to increase HIV and syphilis testing among MSM, our findings demonstrate that HIV and syphilis testing rates remain low among MSM in China. Given that there exists a range of activities related to community engagement in sexual health, we constructed a brief engagement scale using factor analysis and found that greater engagement was associated with higher likelihood of HIV and syphilis testing. Studies conducted in countries with a strong civil society suggest community engagement may expand HIV testing among key populations [20,22,23]. Our findings extend existing literature by focusing on community engagement in sexual health within a limited civil society setting and creating a new engagement metric.

We found a high rate of community engagement in sexual health, with three-quarters of men having a moderate or substantial level of engagement. Studies conducted among Internet-using MSM in Taiwan [34] and non-urban MSM in Canada [37] found lower rates of engagement than that of our study. Additionally, we found MSM with greater community engagement in sexual health were more likely to have ever received HIV and syphilis testing. Previous studies in Kenya [32] and Taiwan [34] showed that community-level involvement was associated with HIV testing. Our results align with those findings, and also suggest that gains in community engagement could linearly translate into HIV testing expansion. The quarter of individuals who had no or minimal engagement suggests the need for more widespread community engagement interventions.

We found that roughly half of MSM in our sample had ever tested for HIV in their lifetime. This figure is similar to MSM data from China, higher than those from Thailand [38], and lower than those from Cambodia [39], South Africa [40], Peru [41], Australia [42], and the US [43]. We also found that only 23% of men tested for HIV at least once every six months, which is much lower than recommendations by US CDC guidelines [36]. Given our sample had engaged in recent condomless sex, a risk behaviour known to be associated with increased HIV testing [44], the low testing rates and frequency in our sample are particularly concerning. In addition, pre-exposure prophylaxis (PrEP) is not available to most MSM in China [45], highlighting the need for strengthening other HIV prevention tools. Current testing efforts are not adequately reaching Chinese MSM and new approaches are urgently needed to address this missed opportunity. A recent modelling study demonstrated that a four-fold increase in testing rates in China may prevent as many as 42,000 HIV infections and 11,000 deaths over the next 5 years [46].

Our study suggests that only one-third of participants had ever tested for syphilis. Our figure corresponds to another study on MSM and transgender individuals in China, which found the syphilis testing rate among MSM to be 31.2% [11]. This is also similar to data from MSM in the US [47]. The low syphilis testing rates among our sample is particularly concerning because both condomless sex [48–50] and syphilis infection [51] increase the risk of HIV transmission. The development of dual rapid test kits for HIV and syphilis [52,53] provides one way to promote increased testing of both within key populations.

Our findings have important implications for strengthening HIV prevention among key populations. Interventions that aim to expand HIV testing should incorporate community-level components that engage MSM, such as online discussions or in-person events related to HIV/STI testing or sexual health. Active, in-person engagements should particularly be encouraged to maximize the potential increase in testing. Community engagement in sexual health may increase testing through creating positive social norms towards testing among peer groups [24–26]. Ways to build community engagement include crowdsourcing, which can shift tasks from individuals to a larger group during intervention development [54,55], and social media, which can serve as a platform to reach multiple social networks during intervention delivery [56]. Community-based organizations (CBOs), despite their limited capacity in China, may be well positioned to build community engagement. A recent study in Taiwan found involvement in AIDS service organizations was associated with community engagement [33]. In China, partnership between CBOs and public sector agencies also demonstrated success in providing MSM with HIV-related services that could be scaled up [57].

Our study has several limitations. First, due to self-reporting of personal information, reporting bias is a concern. However, participation in the online survey was self-administered and anonymous. Second, our survey participants consisted of an online convenience sample of younger MSM populations. At the same time, the online format may have allowed us to collect information from hard-to-reach populations that do not frequent traditional survey sites such as hospitals and clinics. Third, our Cronbach’s alpha for the combination of six community engagement in sexual health items (0.67) was less than the generally acceptable level of 0.70. Fourth, our cross-sectional design makes it difficult to determine temporal associations. Since we cannot temporally separate testing uptake from community engagement in sexual health, we cannot rule out alternative explanations. While our analyses of lifetime HIV testing and lifetime syphilis testing showed the same patterns of association as those in recent HIV testing, it is plausible that lifetime testing could subsequently result in increased engagement. Fifth, we only examined HIV and syphilis testing, but testing for other STIs may be useful for informing public health interventions. Finally, the lack of a consensus on how to define and measure community engagement may complicate comparisons across studies. Some studies measured awareness of CBO activities in the community [32,58] while others measured participation in HIV-related community events [33,37]. Although measurement of a range of community engagement levels has been suggested [25], there is still a need for harmonization of definitions.

## Conclusions

This study demonstrates that greater level of community engagement in sexual health is associated with increased HIV and syphilis testing among MSM in China. We also found that HIV and syphilis testing rates are alarmingly low in this key population. New intervention models are urgently needed and should consider incorporating community engagement components, such as encouraging discussions about or in-person events related to sexual health. Given that there are no specific guidelines for HIV testing frequency among Chinese MSM, clearer guidelines for doctors and public health professionals may also improve testing uptake and frequency. Finally, more research on community engagement is needed to explore how it may improve intervention development and delivery among key populations.

## Notes

To access the supplementary material to this article please see Supplementary Files under Article Tools online.

## References

[1] Choi KH, Lui H, Guo Y, Han L, Mandel JS. Lack of HIV testing and awareness of HIV infection among men who have sex with men, Beijing, China. AIDS Educ Prev. 2006;18(1):33–10. 16539574 10.1521/aeap.2006.18.1.33

[2] Fay H, Baral SD, Trapence G, Motimedi F, Umar E, Iipinge S, et al Stigma, health care access, and HIV knowledge among men who have sex with men in Malawi, Namibia, and Botswana. AIDS Behav. 2011;15(6):1088–97. 21153432 10.1007/s10461-010-9861-2

[3] van Griensven F, de lind van Wijngaarden JW, Baral S, Grulich A The global epidemic of HIV infection among men who have sex with men. Curr Opin HIV AIDS. 2009;4(4):300–7. 19532068 10.1097/COH.0b013e32832c3bb3

[4] Arreola SHP, Makofane K, Beck J, Ayala G Access to HIV prevention and treatment for men who have sex with men: findings from the 2012 Global Men’s Health and Rights survey (GMHR). Oakland, CA: MSM Global Forum; 2012.

[5] Adam PC, de Wit JB, Toskin I, Mathers BM, Nashkhoev M, Zablotska I, et al Estimating levels of HIV testing, HIV prevention coverage, HIV knowledge, and condom use among men who have sex with men (MSM) in low-income and middle-income countries. J Acquir Immune Defic Syndr. 2009;52(Suppl 2):S143–51. 19901627 10.1097/QAI.0b013e3181baf111

[6] Charlebois ED, Das M, Porco TC, Havlir DV The effect of expanded antiretroviral treatment strategies on the HIV epidemic among men who have sex with men in San Francisco. Clin Infect Dis. 2011;52(8):1046–9. 21460322 10.1093/cid/cir085 PMC3070031

[7] Granich RM, Gilks CF, Dye C, De Cock KM, Williams BG Universal voluntary HIV testing with immediate antiretroviral therapy as a strategy for elimination of HIV transmission: a mathematical model. Lancet. 2009;373(9657):48–57. 19038438 10.1016/S0140-6736(08)61697-9

[8] Pineirua A, Sierra-Madero J, Cahn P, Guevara Palmero RN, Martinez Buitrago E, Young B, et al The HIV care continuum in Latin America: challenges and opportunities. Lancet Infect Dis. 2015;15(7):833–9. 26122456 10.1016/S1473-3099(15)00108-5

[9] Chow EP, Lau JT, Zhuang X, Zhang X, Wang Y, Zhang L HIV prevalence trends, risky behaviours, and governmental and community responses to the epidemic among men who have sex with men in China. Biomed Res Int. 2014;2014:607261. 24822214 10.1155/2014/607261 PMC4005141

[10] Zou H, Hu N, Xin Q, Beck J HIV testing among men who have sex with men in China: a systematic review and meta-analysis. AIDS Behav. 2012;16(7):1717–28. 22677975 10.1007/s10461-012-0225-y

[11] Best J, Tang W, Zhang Y, Han L, Liu F, Huang S, et al Sexual behaviors and HIV/syphilis testing among transgender individuals in China: implications for expanding HIV testing services. Sex Transm Dis. 2015;42(5):281–5. 25868142 10.1097/OLQ.0000000000000269 PMC4396656

[12] Chow EP, Wilson DP, Zhang L The rate of HIV testing is increasing among men who have sex with men in China. HIV Med. 2012;13(5):255–63. 22252151 10.1111/j.1468-1293.2011.00974.x

[13] Chow EP, Wilson DP, Zhang L HIV and syphilis co-infection increasing among men who have sex with men in China: a systematic review and meta-analysis. PLoS One. 2011;6(8):e22768. 21857952 10.1371/journal.pone.0022768 PMC3156129

[14] Wu Z, Xu J, Liu E, Mao Y, Xiao Y, Sun X, et al HIV and syphilis prevalence among men who have sex with men: a cross-sectional survey of 61 cities in China. Clin Infect Dis. 2013;57(2):298–309. 23580732 10.1093/cid/cit210 PMC3689345

[15] Tucker JD, Wong FY, Nehl EJ, Zhang F HIV testing and care systems focused on sexually transmitted HIV in China. Sex Transm Infect. 2012;88(2):116–9. 22345024 10.1136/sextrans-2011-050135 PMC3627180

[16] Bien CH, Muessig KE, Lee R, Lo EJ, Yang LG, Yang B, et al HIV and syphilis testing preferences among men who have sex with men in South China: a qualitative analysis to inform sexual health services. PLoS One. 2015;10(4):e0124161. 25875336 10.1371/journal.pone.0124161 PMC4395264

[17] Fan EL HIV testing as prevention among MSM in China: the business of scaling-up. Glob Public Health. 2014;9(1–2):85–97. 24498955 10.1080/17441692.2014.881520

[18] Liu Y, Sun X, Qian HZ, Yin L, Yan Z, Wang L, et al Qualitative assessment of barriers and facilitators of access to HIV testing among men who have sex with men in China. AIDS Patient Care STDS. 2015;29(9):481–9. 26186029 10.1089/apc.2015.0083 PMC4553375

[19] Wei C, Yan H, Yang C, Raymond HF, Li J, Yang H, et al Accessing HIV testing and treatment among men who have sex with men in China: a qualitative study. AIDS Care. 2014;26(3):372–8. 23909807 10.1080/09540121.2013.824538 PMC4053186

[20] Trapence G, Collins C, Avrett S, Carr R, Sanchez H, Ayala G, et al From personal survival to public health: community leadership by men who have sex with men in the response to HIV. Lancet. 2012;380(9839):400–10. 22819662 10.1016/S0140-6736(12)60834-4 PMC3805044

[21] CDC/ATSDR Committee on Community Engagement Principles of community engagement. Atlanta, GA: Center for Disease Control and Prevention; 1997.

[22] Young SD, Cumberland WG, Nianogo R, Menacho LA, Galea JT, Coates T The HOPE social media intervention for global HIV prevention in Peru: a cluster randomised controlled trial. Lancet HIV. 2015;2(1):e27–32. 26236767 10.1016/S2352-3018(14)00006-X PMC4520433

[23] Coates TJ, Kulich M, Celentano DD, Zelaya CE, Chariyalertsak S, Chingono A, et al Effect of community-based voluntary counselling and testing on HIV incidence and social and behavioural outcomes (NIMH Project Accept; HPTN 043): a cluster-randomised trial. Lancet Glob Health. 2014;2(5):e267–77. 25103167 10.1016/S2214-109X(14)70032-4 PMC4131207

[24] Meyer IH Prejudice, social stress, and mental health in lesbian, gay, and bisexual populations: conceptual issues and research evidence. Psychol Bull. 2003;129(5):674–97. 12956539 10.1037/0033-2909.129.5.674 PMC2072932

[25] Ramirez-Valles J The protective effects of community involvement for HIV risk behavior: a conceptual framework. Health Educ Res. 2002;17(4):389–403. 12197585 10.1093/her/17.4.389

[26] Latkin C, Weeks MR, Glasman L, Galletly C, Albarracin D A dynamic social systems model for considering structural factors in HIV prevention and detection. AIDS and Behavior. 2010;14(Suppl 2):222–38. 20838871 10.1007/s10461-010-9804-y PMC3006194

[27] The World Bank Defining Civil Society 2013. [cited 2016 524] Available from: http://go.worldbank.org/4CE7W046K0

[28] Li H, Kuo NT, Liu H, Korhonen C, Pond E, Guo H, et al From spectators to implementers: civil society organizations involved in AIDS programmes in China. Int J Epidemiol. 2010;39(Suppl 2):ii65–71. 21113039 10.1093/ije/dyq223 PMC2992623

[29] Sullivan PS, Carballo-Dieguez A, Coates T, Goodreau SM, McGowan I, Sanders EJ, et al Successes and challenges of HIV prevention in men who have sex with men. Lancet. 2012;380(9839):388–99. 22819659 10.1016/S0140-6736(12)60955-6 PMC3670988

[30] Grover E, Grosso A, Ketende S, Kennedy C, Fonner V, Adams D, et al Social cohesion, social participation and HIV testing among men who have sex with men in Swaziland. AIDS Care. 2016;28(6):795–804. 26824888 10.1080/09540121.2015.1131971

[31] Chuncheng Liu TW, Mao J, Tang W, Tso LS, Tang S, Zhang W, et al Crowdsourcing versus social marketing video campaigns to promote condom use: a non-inferiority randomized controlled trial among MSM and transgender individuals in China. Hong Kong: International Behavioral Health Conference; 2016 2016 115 Report No.: O12b.

[32] Riehman KS, Kakietek J, Manteuffel BA, Rodriguez-Garcia R, Bonnel R, N’Jie N, et al Evaluating the effects of community-based organization engagement on HIV and AIDS-related risk behavior in Kenya. AIDS Care. 2013;25(Suppl 1):S67–77. 23745632 10.1080/09540121.2013.778383 PMC4003576

[33] Chuang DM, Lacombe-Duncan A Community engagement among men who have sex with men living with HIV/AIDS in Taiwan. AIDS Care. 2016;28(4):445–9. 26586156 10.1080/09540121.2015.1112355

[34] Ko NY, Hsieh CH, Wang MC, Lee C, Chen CL, Chung AC, et al Effects of Internet popular opinion leaders (iPOL) among Internet-using men who have sex with men. J Med Internet Res. 2013;15(2):e40. 23439583 10.2196/jmir.2264 PMC3636233

[35] Suhr DD Proceedings of the thirty-first annual SAS users group international conference: exploratory or confirmatory factor analysis? Cary, NC:SAS Institute Inc;2006 Contract No.: Paper 200-31.

[36] Workowski KA, Bolan GA, Centers for Disease C, Prevention Sexually transmitted diseases treatment guidelines, 2015. MMWR Recomm Rep. 2015;64(RR–03):1–137. PMC5885289 26042815

[37] Holtzman S, Landis L, Walsh Z, Puterman E, Roberts D, Saya-Moore K Predictors of HIV testing among men who have sex with men: a focus on men living outside major urban centres in Canada. AIDS Care. 2016;28(6):705–11. 27043184 10.1080/09540121.2016.1164288

[38] Sapsirisavat V, Phanuphak N, Keadpudsa S, Egan JE, Pussadee K, Klaytong P, et al Psychosocial and behavioral characteristics of high-risk men who have sex with men (MSM) of unknown HIV positive serostatus in Bangkok, Thailand. AIDS and Behavior. 2016;20(Suppl 3):386–97. 27553027 10.1007/s10461-016-1519-2

[39] Yi S, Tuot S, Chhoun P, Brody C, Pal K, Oum S Factors associated with recent HIV testing among high-risk men who have sex with men: a cross-sectional study in Cambodia. BMC Public Health. 2015;15:743. 26231524 10.1186/s12889-015-2096-4 PMC4522123

[40] Lane T, Osmand T, Marr A, Shade SB, Dunkle K, Sandfort T, et al The Mpumalanga Men’s Study (MPMS): results of a baseline biological and behavioral HIV surveillance survey in two MSM communities in South Africa. PLoS One. 2014;9(11):e111063. 25401785 10.1371/journal.pone.0111063 PMC4234301

[41] Lee SW, Deiss RG, Segura ER, Clark JL, Lake JE, Konda KA, et al A cross-sectional study of low HIV testing frequency and high-risk behaviour among men who have sex with men and transgender women in Lima, Peru. BMC Public Health. 2015;15:408. 25896917 10.1186/s12889-015-1730-5 PMC4407786

[42] de Wilt J, Mao L, Adam P, Treloar C HIV/AIDS, hepatitis and sexually transmissible infections in Australia: annual report of trends in behaviour 2015. Sydney: Centre for Social Research in Health, UNSW Australia; 2015.

[43] Sanchez T, Zlotorzynska M, Sineath C, Kahle E, Sullivan P The annual American men’s internet survey of behaviors of men who have sex with men in the United States: 2014 key indicators report. JMIR Public Health Surveill. 2016;2(1):e23. 27244770 10.2196/publichealth.5476 PMC4909387

[44] Stephenson R, White D, Darbes L, Hoff C, Sullivan P HIV testing behaviors and perceptions of risk of HIV infection among MSM with main partners. AIDS Behav. 2015;19(3):553–60. 25081599 10.1007/s10461-014-0862-4 PMC4314517

[45] Zhang Y, Peng B, She Y, Liang H, Peng HB, Qian HZ, et al Attitudes toward HIV pre-exposure prophylaxis among men who have sex with men in western China. AIDS Patient Care STDS. 2013;27(3):137–41. 23425017 10.1089/apc.2012.0412 PMC3595955

[46] Zhang L, Gray RT, Wilson DP Modelling the epidemiological impact of scaling up HIV testing and antiretroviral treatment in China. Sex Health. 2012;9(3):261–71. 22697144 10.1071/SH11104

[47] Said MA, German D, Flynn C, Linton SL, Blythe D, Cooley LA, et al Uptake of testing for HIV and syphilis among men who have sex with men in Baltimore, Maryland: 2004-2011. AIDS Behav. 2015;19(11):2036–43. 26078117 10.1007/s10461-015-1106-y

[48] Jin F, Jansson J, Law M, Prestage GP, Zablotska I, Imrie JC, et al Per-contact probability of HIV transmission in homosexual men in Sydney in the era of HAART. AIDS. 2010;24(6):907–13. 20139750 10.1097/QAD.0b013e3283372d90 PMC2852627

[49] Patel P, Borkowf CB, Brooks JT, Lasry A, Lansky A, Mermin J Estimating per-act HIV transmission risk: a systematic review. AIDS. 2014;28(10):1509–19. 24809629 10.1097/QAD.0000000000000298 PMC6195215

[50] Darrow WW, Jaffe HW, Curran JW Passive anal intercourse as a risk factor for AIDS in homosexual men. Lancet. 1983;2(8342):160. 10.1016/s0140-6736(83)90139-3 6135002

[51] Stamm WE, Handsfield HH, Rompalo AM, Ashley RL, Roberts PL, Corey L The association between genital ulcer disease and acquisition of HIV infection in homosexual men. JAMA. 1988;260(10):1429–33. 3404600

[52] Bristow CC, Severe L, Pape JW, Javanbakht M, Lee SJ, Comulada WS, et al Dual rapid lateral flow immunoassay fingerstick wholeblood testing for syphilis and HIV infections is acceptable and accurate, Port-au-Prince, Haiti. BMC Infect Dis. 2016;16(1):302. 27316352 10.1186/s12879-016-1574-3 PMC4912739

[53] Yin YP, Ngige E, Anyaike C, Ijaodola G, Oyelade TA, Vaz RG, et al Laboratory evaluation of three dual rapid diagnostic tests for HIV and syphilis in China and Nigeria. Int J Gynaecol Obstet. 2015;130(Suppl 1):S22–6. 25975869 10.1016/j.ijgo.2015.04.004

[54] Tang W, Han L, Best J, Zhang Y, Mollan K, Kim J, et al Crowdsourcing HIV test promotion videos: a noninferiority randomized controlled trial in China. Clin Infect Dis. 2016;62(11):1436–42. 27129465 10.1093/cid/ciw171 PMC4872295

[55] Zhang Y, Kim JA, Liu F, Tso LS, Tang W, Wei C, et al creative contributory contests to spur innovation in sexual health: 2 cases and a guide for implementation. Sex Transm Dis. 2015;42(11):625–8. 26462186 10.1097/OLQ.0000000000000349 PMC4610177

[56] Tso LS, Tang W, Li H, Yan HY, Tucker JD Social media interventions to prevent HIV: a review of interventions and methodological considerations. Curr Opin Psychol. 2016;9:6–10. 26516632 10.1016/j.copsyc.2015.09.019 PMC4620570

[57] Cheng W, Cai Y, Tang W, Zhong F, Meng G, Gu J, et al Providing HIV-related services in China for men who have sex with men. Bull World Health Organ. 2016;94(3):222–7. 26966334 10.2471/BLT.15.156406 PMC4773928

[58] Dangerfield DT 2nd, Gravitt P, Rompalo AM, Yap I, Tai R, Lim SH Awareness and utilization of HIV services of an AIDS community-based organization in Kuala Lumpur, Malaysia. Int J STD AIDS. 2015;26(1):20–6. 24676132 10.1177/0956462414528685

